# Current Management of Infective Endocarditis: A Narrative Review Focused on Unmet Clinical Needs and the Multidisciplinary Approach

**DOI:** 10.3390/jcdd13040155

**Published:** 2026-03-30

**Authors:** Luca Di Vito, Giuseppina D’Amato, Riccardo Pascucci, Antonella D’Antonio, Giancarla Scalone, Mariavirginia Boni, Brunella Rossi, Ilaria Cimaroli, Claudia Acciarri, Marida Andreucci, Andrea Romandini, Simona Silenzi, Procolo Marchese, Pierfrancesco Grossi

**Affiliations:** 1 Cardiology Unit, C. and G. Mazzoni Hospital, AST Ascoli Piceno, 63100 Ascoli Piceno, Italy; 2 Infectious Disease Unit, C. and G. Mazzoni Hospital, AST Ascoli Piceno, 63100 Ascoli Piceno, Italy; 3 Internal Medicine Unit, C. and G. Mazzoni Hospital, AST Ascoli Piceno, 63100 Ascoli Piceno, Italy; 4 Nuclear Medicine Unit, C. and G. Mazzoni Hospital, AST Ascoli Piceno, 63100 Ascoli Piceno, Italy

**Keywords:** infective endocarditis, *Staphylococcus aureus*, prosthetic heart valve, intracardiac device, nuclear medicine, echocardiography

## Abstract

Infective endocarditis (IE) is a severe infectious disease affecting cardiac valves (either native or prosthetic) or implantable cardiac devices, and it is associated with high rates of morbidity and mortality. Recent data from the Global Burden of Disease study have shown a significant increase in both the incidence and mortality of IE. One-year mortality following diagnosis can reach up to 30%. IE can present with a wide range of clinical manifestations, and its course may be complicated by systemic embolic events or intracardiac complications such as abscess formation or prosthetic valve dehiscence. Echocardiography remains the first-line imaging modality; however, an integrated multimodality imaging approach is increasingly adopted in contemporary practice, incorporating both cardiac computed tomography and positron emission tomography. A multidisciplinary approach involving cardiologists, cardiac surgeons, internists, infectious disease specialists, and nuclear medicine physicians is often required to ensure accurate diagnosis and effective treatment of IE. The prognosis of infective endocarditis depends on early diagnosis, appropriate antimicrobial therapy, and timely surgical intervention when indicated. This review aims to summarize the current knowledge on IE, from pathophysiological insights to surgical strategies. It also focuses on practical recommendations to address the most pressing unmet clinical needs through a multidisciplinary approach.

## 1. Introduction

### 1.1. Epidemiology and Current Trends in Incidence and Mortality

Infective endocarditis (IE) is a severe infection involving the cardiac valves, either native or prosthetic, the endocardium, or intracardiac devices. An analysis from the global burden of disease study, which included IE data from 204 countries, revealed a global increase in the burden of IE, primarily driven by a rise in the age-standardized incidence rate from 9 cases per 100,000 population in 1990 to 12 cases per 100,000 in 2021 [1]. A subanalysis restricted to high-income European countries showed a more recent plateau in incidence trends, with some exceptions indicating a continued increase in the United Kingdom and Germany [1].

Between 1990 and 2019, the mortality rate related to IE increased markedly, particularly in Italy [1]. However, during the most recent period (2013–2019), a stabilization or even a slight decline in mortality has been observed.

This trend has been attributed to improvements in the management of IE, including the use of new antibiotics and novel antimicrobial combinations, publication of updated guidelines, the increasing proportion of patients undergoing surgery, and the increasing establishment of dedicated multidisciplinary teams (MDTs) especially in referring hospitals [2,3]. However, recent data shows that MDTs are still lacking in most hospitals, especially where cardiac surgery is not available on site [4]. This represents a current unmet need.

A retrospective analysis of 17,407 cases of IE from 963 hospitals across Japan between 2016 and 2021 showed an increasing proportion of patients aged over 80 years, rising from 25 percent to 31 percent, together with a higher prevalence of atrial fibrillation and overall patient frailty [5]. The study also reported a progressive decline in the proportion of patients with pre-existing valvular disease. The authors attributed these findings to the growing incidence of healthcare-associated IE, particularly among patients without prior known structural valvular abnormalities [5].

In lines with these finding, another retrospective study conducted between 1997 and 2014 in Spain demonstrated a progressive decline in community-acquired IE from 60% to 49%, accompanied by a simultaneous increase in nosocomial IE from 40% to 52% [6]. Consequently, the overall rise in IE incidence can largely be attributed to healthcare-associated factors such as the widespread use of indwelling catheters, prosthetic materials, and invasive procedures [6].

At present, native valve endocarditis (NVE) remains the most common form (56.6%), followed by prosthetic valve endocarditis (PVE) (30.1%) and cardiac device-related infective endocarditis (CDRIE) (9.9%) [7].

### 1.2. Typical Clinical Findings According to EURO-ENDO Registry Results

According to the EURO-ENDO registry [7], which enrolled 3116 patients with IE from 40 countries, including 11.7% with congenital heart disease, the most common clinical findings at presentation were fever (77.7%) and cardiac murmur (64.5%), followed by congestive heart failure (27.2%) and septic shock (6.6%). Cardiogenic shock occurred in 2.3% of patients with IE. Janeway lesions, Osler nodes, and Roth spots were less frequently observed, occurring in 1.5–3.5% of cases, the majority of which were reported in patients with NVE.

Septic embolic events were detected in 25.3% of patients with IE at presentation, with notable differences in localization between PVE and NVE versus CDRIE. Cerebral and splenic embolization were more frequent in PVE and NVE, whereas pulmonary emboli predominated in CDRIE. Most cerebral embolic events were asymptomatic, accounting for 55.9% of cases, while hemorrhagic stroke occurred in 2.2% of patients. Mycotic aneurysms were identified in 1.9% of patients. Hepatic emboli were more frequent in CDRIE despite ongoing medical therapy. Perivalvular abscesses were more common in PVE (13.8%) compared with NVE (11.5%) and CDRIE (7.8%). Finally, spondylitis was reported in 5.4% of patients with IE.

Positive blood cultures were obtained in 79% of IE patients. Positive rheumatoid factor was detected in 17% of EI cases (mostly NVE). The most frequently isolated microorganisms were staphylococci in 44.1%, enterococci in 15.8%, oral streptococci in 12.4%, *Streptococcus gallolyticus* in 6.6%, and Gram-negative bacilli in 3.5%. *Coxiella burnetii* IE was identified in 0.8%.

Methicillin-resistant *Staphylococcus aureus* (MRSA, 8.4%) and viridans group streptococci (15.1%) were the most frequent pathogens in NVE, whereas methicillin-resistant coagulase-negative staphylococci (CoNS) (10.2%) and enterococci (21.7%) were more common in PVE. Both methicillin-sensitive *Staphylococcus aureus* (MSSA) and methicillin-sensitive CoNS were predominant in CDRIE (39.5% and 12%, respectively).

A distinct microbiological profile has been reported in studies focusing on IE after transcatheter aortic valve implantation (TAVI). In a dedicated TAVI-IE cohort, *Enterococcus* species (spp.) accounted for approximately 35% of cases, while *Streptococcus gallolyticus* and other streptococci of suspected gastrointestinal origin represented about 18% of infections. Among enterococci, *Enterococcus faecalis* was the most common cause of IE, owing to specific virulence factors that promote epithelial cell adhesion and enhance biofilm formation [8].

In addition, more than half of TAVI-related IE episodes (54%) were classified as healthcare-associated or nosocomial infections, highlighting the prominent role of peri-procedural and in-hospital exposure in this setting [9].

Adverse clinical events during hospitalization were observed in a substantial proportion of patients with IE. Acute renal failure occurred in 17.5% of cases, particularly among those with PVE and CDRIE. Persistent fever lasting more than seven days was reported in 12.4% of patients, while positive blood cultures after 48 h of antibiotic therapy were documented in 13.4%. Atrioventricular block developed in 4.5% of cases, and thrombocytopenia was observed in 7.6% [7].

## 2. Pathophysiology of Infectious Endocarditis: Focus on the *Staphylococcus aureus*

### 2.1. Overview of Staphylococcus aureus Virulence and Initial Adhesion Mechanisms

The pathophysiology of IE is multifactorial and involves a dynamic interplay between host immune responses, endothelial activation or damage, and the microbiological characteristics of the invading pathogen. Among all causative microorganisms, *Staphylococcus aureus* (SA) represents the most virulent species and is consistently associated with the worst clinical outcomes, including higher mortality and a substantially increased risk of embolic events. SA functions as a master manipulator of host biology, exploiting both immune and coagulation pathways to promote adhesion, persistence, and dissemination within the cardiovascular system [10]. SA possesses a unique set of virulence traits that allow efficient adhesion to cardiac valves despite the high-flow and high-shear-stress environment that normally prevents bacterial attachment [11]. This shear-enhanced adhesion provides SA with a substantial competitive advantage, enabling it to colonize sites that are typically resistant to microbial adherence [11]. Once initial attachment occurs, SA rapidly forms a biofilm, a structured formation embedded in an extracellular matrix composed of polysaccharides, surface proteins, lipoteichoic acids, and extracellular DNA [12]. Biofilm development is a critical virulence determinant in IE because it anchors bacterial aggregates within fibrin-platelet thrombi, protects them from mechanical forces, and provides an immunologically privileged niche. The biochemical complexity and density of the matrix profoundly modify antibiotic efficacy [12]. Antibiotics that appear highly active in vitro often fail to eradicate infection in vivo because the matrix impedes penetration, neutralizes antimicrobial molecules, and shelters dormant bacteria, which are intrinsically less responsive to cell wall-active or metabolism-dependent agents. This disparity explains why clinical clearance often lags microbiological expectations and contributes to treatment failure, relapse, and the persistence of viable embolic fragments [12].

SA further manipulates the local hemostatic environment through coagulases and staphylokinase, two factors whose effects depend on bacterial density [13]. Coagulases convert fibrinogen to fibrin independently of the host coagulation cascade, promoting rapid encasement of bacterial clusters and stabilizing the nascent vegetation. As bacterial biomass increases, SA produces staphylokinase, which triggers localized fibrinolysis and activates host metalloproteases. This sequential switch, from fibrin deposition to partial fibrinolysis, facilitates remodeling of the infected thrombus, loosening of surface layers, and the release of biofilm-coated emboli capable of systemic dissemination [14].

### 2.2. Dual Pathophysiological Pathways: Endothelial Damage Versus Endothelial Activation

Two distinct pathophysiological mechanisms have been described for SA IE [15,16]. The first mechanism is related to endothelial damage, as observed in congenital heart disease, prosthetic material, or degenerative valve disease. Detachment of the endothelial layer exposes the subendothelial matrix, which is rich in fibrin, fibronectin, collagen, and von Willebrand factor (VWF). This environment allows direct adhesion of SA and promotes stable bacterial attachment and rapid vegetation development, with platelets playing only a marginal role in the initial stages [15,16].

In contrast, the second mechanism involves endothelial inflammation or activation, triggered by systemic sepsis or chronic inflammatory stimuli such as intravenous (IV) drug use [15,16]. Under these conditions, endothelial cells release ultra-large VWF multimers and actively recruit platelets to the luminal surface. In this setting, SA adhesion relies predominantly on platelet-mediated interactions, facilitating rapid colonization even in the absence of structural endothelial injury [15,16].

The consequences of these two distinct mechanisms are clinically relevant. In SA IE driven by endothelial damage, the role of platelets is minimal, which explains the limited efficacy of antiplatelet agents in preventing IE [15,16]. Conversely, in SA IE occurring on inflamed or activated endothelium, the redundancy of platelet-bacteria interactions contribute to the modest effectiveness of SA vaccination strategies and antibiotic prophylaxis, since bacterial adhesion can occur through multiple alternative pathways that bypass the targeted mechanisms [15,16].

## 3. Microbiology of Infective Endocarditis

The microbiology of IE is heterogenous and depends on host-related factors, underlying structural heart disease, healthcare exposure, and regional epidemiological patterns [17,18]. Gram-positive cocci remain the predominant causative agents worldwide.

### 3.1. Gram-Positive Cocci

SA has emerged as the leading pathogen in many settings, particularly in-hospital-acquired infections, affecting both native and prosthetic valves. MRSA continues to pose a major challenge due to limited antibiotic susceptibility and worse outcome [19].

CoNS, most notably Staphylococcus epidermidis, are frequently implicated in PVE and CDRIE due to their ability to adhere to foreign material through biofilm formation [17,20].

Streptococci, particularly viridans group streptococci, remain a common cause of community-acquired IE and are typically associated with subacute clinical courses involving native valves. These organisms are often linked to transient bacteremia of oral origin. *Streptococcus gallolyticus* requires specific attention because of its association with colorectal neoplasia which warrants systematic gastrointestinal evaluation upon detection [17,21].

Enterococci, predominantly Enterococcus faecalis, represent an increasingly relevant etiological group, especially among elderly patients and those with repeated healthcare contact. Enterococcal IE is commonly associated with genitourinary or gastrointestinal procedures and is characterized by intrinsic resistance mechanisms [17,22].

### 3.2. Gram-Negative Bacteria and Other Pathogens

Gram-negative organisms are less frequent causes of IE. The HACEK group (*Haemophilus* spp., *Aggregatibacter* spp., *Cardiobacterium hominis*, *Eikenella corrodens*, and *Kingella* spp.) is associated with a subacute course and is historically linked to culture-negative endocarditis [17,23].

Non-HACEK Gram-negative bacilli are rare causes of IE and most often encountered in IV drug users or healthcare-associated infections [18,24].

### 3.3. Fungal Endocarditis

Fungal IE remains an infrequent but highly lethal entity. It predominantly affects immunocompromised individuals, IV drug users, and patients with prosthetic valves or intracardiac devices [18,25]. Candida species are the most identified pathogens, whereas Aspergillus species often cause culture-negative disease.

### 3.4. Blood Culture-Negative Endocarditis

Blood culture-negative IE continues to represent a significant diagnostic challenge. This condition may result from prior antibiotic exposure or infection with fastidious or intracellular organisms, including *Coxiella burnetii*, *Bartonella* spp., and the HACEK group [21,26]. Molecular techniques, such as brad-range 16S rRNA polymerase chain reaction applied to excised valve specimens obtained during cardiac surgery, have improved pathogen detection [26].

### 3.5. Trends in Antimicrobial Resistance and Clinical Implications

Antimicrobial resistance has become an important clinical concern, especially for IE due to MRSA [19]. Enterococci may also exhibit high-level resistance to aminoglycoside due to both intrinsic and acquired mechanisms [27].

## 4. Laboratory and Microbiological Work-Up

### Blood Cultures: Timing, Repetition, and Pathogen Identification

Blood cultures represent the fundamental diagnostic tool for bloodstream infections. Current recommendations advise collecting two to three blood culture sets from different venipuncture sites, ideally prior to antibiotic administration in order to maximize diagnostic yield and to differentiate true bacteremia from contamination [17,18].

The integration of rapid diagnostic technologies, such as matrix-assisted laser desorption/ionization time-of-flight mass spectrometry (MALDI-TOF MS) has substantially shortened pathogen identification time [20,28].

Molecular diagnostic approaches, such as polymerase chain reaction (PCR)-based methods applied on blood sample or excised tissue, allow direct detection of microbial nucleic acids and can be used in cases of blood culture-negative IE [23,25]. However, multiplex molecular assays have a limited ability to detect less common pathogens such as those of the HACEK group, while demonstrating high accuracy for common organisms, particularly streptococci [29]. Serological assays can contribute to diagnosis of IE caused by intracellular pathogens such as *Coxiella burnetii* or *Bartonella* species [30]. Inflammatory biomarkers such as C-reactive protein (CRP) and procalcitonin may support the diagnosis of IE and are associated with infection severity and microbial burden [31,32].

## 5. Diagnostic Criteria and Classifications

Diagnosis of IE relies on a composite framework that integrates microbiology, imaging, and clinical context, with pathological confirmation remaining the reference standard when available.

The original Duke criteria [33] classified IE as definite, possible, or rejected based on the presence of major and minor criteria.

The modified Duke criteria [34] represented a pivotal refinement of the original framework by formalizing the definition of persistent bacteraemia and incorporating *Coxiella burnetii* serology as a major criterion, thereby substantially improving diagnostic reproducibility.

Subsequently, both the European Society of Cardiology (ESC), through its 2015 [35] and 2023 [36] diagnostic criteria, and the International Society for Cardiovascular Infectious Diseases (ISCVID), with the 2023 update [37], modified the original major/minor framework to accommodate advances in imaging technology, evolving microbiological knowledge, and the increasing prevalence of intracardiac prosthetic material and cardiac implantable electronic devices (Table 1).

Across clinical settings, newer diagnostic criteria generally increase sensitivity at the expense of reduced specificity, with the overall balance strongly influenced by pre-test probability and the population under evaluation. In a large prospective cohort of patients treated for IE, diagnostic performance differed markedly in CDRIE. Among patients with a intracardiac device, both the 2023 Duke-ISCVID criteria and the 2023 ESC criteria demonstrated very high sensitivity (97.1% and 93.8%, respectively) but lower specificity (31.1% and 41.0%, respectively), as compared with the 2015 ESC criteria (62.3%). Specificity declined even further in the subgroup with lead-related IE, highlighting how expanded microbiological and pathological definitions, together with broader imaging triggers, may inflate “definite” classifications when lead infection is suspected [38].

By contrast, in patients without CDRIE, representing a predominantly PVE population, sensitivities remained uniformly high (>96%) across the 2015 ESC, 2023 Duke-ISCVID, and 2023 ESC criteria, whereas specificity was moderate and showed a stepwise decline from 55.5% with the 2015 ESC criteria to 52.6% with the 2023 Duke-ISCVID criteria and 48.0% with the 2023 ESC criteria [38].

PVE remains diagnostically challenging, and the incremental value of updated diagnostic criteria is largely driven by advances in cardiac imaging. In a cohort focusing on TAVI related IE, the original Duke criteria showed limited sensitivity (65%) despite excellent specificity (100%). The incorporation of imaging criteria in the 2015 ESC classification increased sensitivity to 73%, with an expected reduction in specificity (90%). The 2023 updates further improved sensitivity (ISCVID 2023: 76%; ESC 2023: 77%) while maintaining specificity at 90%, underscoring the growing role of multimodality imaging in the evaluation of prosthetic material [9]. In particular, ^18^F-fluorodeoxyglucose positron emission tomography combined with computed tomography (FDG-PET/CT) enabled correct reclassification of patients as having definite IE who were previously excluded under the ESC 2015 criteria, whereas cardiac computed tomography (CT) angiography showed a more limited incremental diagnostic contribution [9].

In enterococcal bacteraemia, which is associated with an increased risk of IE, particularly in elderly patients and those undergoing TAVI [8], both 2023 Duke-based classifications showed improved sensitivity for IE compared with the 2015 ESC criteria (79% for Duke-ISCVID 2023 and 74% for ESC 2023 vs. 67% for ESC 2015). However, this gain in sensitivity was accompanied by a marked reduction in specificity for the Duke-ISCVID 2023 criteria (55%) when compared with the ESC 2015 (86%) and ESC 2023 (69%) classifications [8].

Similarly, in episodes of bacteraemia or candidaemia caused by so-called “new typical” microorganisms, the Duke-ISCVID 2023 criteria demonstrated markedly higher sensitivity (57%) compared with both the ESC 2015 (5%) and ESC 2023 (8%) criteria, while maintaining very high specificity (approximately 99–100%) [39]. In this framework, *Staphylococcus lugdunensis* and *Abiotrophia* spp. are considered typical pathogens irrespective of the presence of intracardiac prosthetic material, whereas *Staphylococcus epidermidis* and *Candida* spp. are classified as typical only in patients with prosthetic valves or other intracardiac devices. Among these newly defined typical microorganisms, *Staphylococcus epidermidis* was associated with the highest risk of IE following bacteraemia, occurring in approximately 52% of cases [39].

Taken together, available evidence suggests that 2023 Duke-ISCVID and 2023 ESC criteria improve case capture, especially in PVE/CDRIE contexts and in certain bacteremia profiles, at the potential cost of more “false definite” classifications in high-suspicion populations where intracardiac material is present. Accordingly, criteria should be applied alongside expert clinical judgement and MDT review, with careful attention to the underlying setting (NVE vs. PVE vs. CDRIE), the timing and quality of imaging, and the plausibility of an alternative source for bloodstream infection.

## 6. Cardiac Device-Related Infective Endocarditis

### 6.1. Epidemiology and Microbiology

CDRIE is an increasingly common complication associated with pacemakers and other intracardiac systems. Infection typically results from peri-procedural contamination, most often from the patient’s own skin flora [40], or from subsequent hematogenous seeding [41]. SA and CoNS are the predominant pathogens due to their strong biofilm formation and affinity for prosthetic material [42]. Gram-negative bacteremia uncommonly causes CDRIE [42], and device infection is generally unlikely in these cases unless another source of bacteremia is ruled out. Fungal infections, particularly those caused by Candida species, represent a rare cause of IE, but may occur in immunocompromised patients and are associated with a worse prognosis [43].

Three main categories of CDRIE are recognized [44]:

1. Superficial incisional infections.
2. Isolated pocket infections.
3. Systemic infections with the presence of positive blood cultures. This group is further subdivided based on the presence of vegetations on leads and/or valves.

### 6.2. Diagnosis

Pocket infections may present with erythema, swelling, tenderness, or purulent discharge, but local signs may be absent. A positive blood culture, particularly SA, significantly increases the likelihood of CDRIE, even in the absence of vegetations. Persistent bacteremia should always trigger full diagnostic evaluation [44].

Transesophageal echocardiography (TEE) remains the primary imaging tool for identifying lead vegetations, though the absence of visualized vegetations does not exclude CDRIE, as extracardiac lead segments may be involved. TTE alone is not recommended for diagnosis due to low sensitivity.

FDG-PET/CT markedly improve diagnostic sensitivity and specificity and are particularly useful when pocket findings are absent [44]. Limitations exist in cases of recently implanted devices (<6 weeks) [36]. These imaging modalities are incorporated into ESC diagnostic criteria for suspected CDRIE [36].

### 6.3. Prevention

Effective prevention includes pre-procedural treatment of active infections [45], glycemic optimization [46], and selective decolonization [47]. Perioperative antibiotic prophylaxis includes IV cefazolin or vancomycin in β-lactam-allergic patients or high MRSA prevalence settings [36,44].

Pocket hematoma is one of the most important modifiable risk factors for subsequent CDRIE. Hematoma formation delays wound healing, often requires interruption of antithrombotic therapy, increases the likelihood of reinterventions, and significantly elevates infection risk. Large cohort studies report a clinically significant hematoma incidence of 2.5–7.3%, with a strong and consistent association with postoperative CDRIE [48].

Anticoagulation and antiplatelet therapy are among the strongest predictors of hematoma formation. In a large contemporary cohort, aspirin nearly doubled the risk of clinically significant hematoma, while dual antiplatelet therapy increased the risk almost four-fold (adjusted OR 3.9) [49]. Heparin bridging further amplified bleeding risk (adjusted OR 2.1) [48]. Similar findings emerged in an Italian multicenter study of 500 patients, where low-molecular-weight heparin bridging showed the highest hematoma rate (11%), and was an independent predictor of hematoma (OR 3.8) [50].

Evidence from randomized trials has reshaped perioperative anticoagulation management. The BRUISE CONTROL trial demonstrated that continuing warfarin during device surgery reduced hematoma formation by 80% compared with heparin bridging, without increasing thromboembolic events [51]. The BRUISE CONTROL-2 trial confirmed that, in direct oral anticoagulant (DOAC)-treated patients, interruption vs. continuation of DOACs produced similar hematoma rates, emphasizing that heparin bridging should be avoided, as it confers unnecessary bleeding risk without clinical benefit [52].

Additional predictors of hematoma include INR ≥ 2, low left ventricular ejection fraction, complex procedures such as cardiac resynchronization therapy with a defibrillator, implantable cardioverter-defibrillator, and the presence of mechanical valve prostheses, which markedly increase bleeding risk [50]. These observations underscore the importance of individualized antithrombotic management.

Preventive strategies targeted to optimize anticoagulation management are summarized in Table 2 [36,44]:

Strict sterile technique and chlorhexidine-alcohol skin preparation remain essential. Antibacterial envelopes, such as the absorbable TYRX™ mesh releasing minocycline and rifampin for ≥7 days and fully resorbed within 9 weeks, reduce major CDRIE by 40% (WRAP-IT trial) [53]. Real-world evidence (REINFORCE) confirms reduced infection rates in high-risk patients [54]. Taurolidine-based agents such as TauroPace™ show promise in early registry data, though randomized trials are needed [55].

### 6.4. Device Removal and CDRIE Management

Delayed treatment significantly worsens prognosis. Antibiotic therapy without device removal is associated with a significant increase in early mortality [56]. Systemic infection carries higher mortality than localized infection; the ELECTRa registry identified systemic presentation as an independent predictor of death [57].

For these reasons, complete system removal without unnecessary delay is strongly recommended.

Percutaneous transvenous extraction is the method of first choice. Vegetations > 20 mm may warrant consideration of open surgical extraction due to embolic risk. In high-volume centers, transvenous extraction has high success and low complication rates, whereas surgery is reserved for large vegetations, extensive valve involvement, or abscess formation [44].

Indications for complete cardiac implantable electronic device (CIED) removal include bacteremia or fungemia with *SA*, CoNS, *Cutibacterium* spp., *Candida* spp., β-hemolytic streptococci, and Enterococcus spp. Device removal is also indicated in cases of recurrent or persistent bacteremia without an alternative source [44].

In bacteremia with non-pseudomonal Gram-negative organisms or pneumococci, device infection is uncommon and removal is generally not required unless persistent [36,44].

### 6.5. Empirical Antimicrobial Therapy

Empirical antibiotic therapy should be initiated after obtaining blood cultures or wound cultures. Once culture results are available, antimicrobial treatment must be adjusted accordingly [36,44].

For superficial incisional infections, oral antibiotic therapy (e.g., amoxicillin-clavulanate) targeting *SA* for 7–10 days is recommended. If MRSA is suspected, an alternative agent active against MRSA should be used [44].

For isolated pocket infections, empirical IV therapy against methicillin-resistant CoNS and SA is recommended (vancomycin or daptomycin). Combination therapy with agents active against Gram-negative bacteria (e.g., a third-generation cephalosporin) is not routinely required, except in the presence of systemic symptoms [44].

In systemic infection, defined as positive blood cultures with or without vegetations on leads or valves and with or without pocket involvement, combination intravenous therapy is mandatory. If prosthetic material is present (e.g., a prosthetic heart valve), rifampicin should be added [44].

Systemic involvement necessitates full IV therapy and precludes any switch to oral antibiotics [44].

After extraction, targeted IV antibiotics are continued for 4–6 weeks [44].

In blood culture-positive CDRIE with lead vegetation, treatment may be shortened to 2 weeks post-extraction if [36,44]:

- TEE after removal shows no valve involvement.
- Blood cultures clear promptly.
- Clinical improvement is rapid.
- No pulmonary abscesses are present.

However, total treatment duration must not be shorter than 4 weeks in systemic infections.

### 6.6. Reimplantation

Re-evaluating the true indication for pacing or defibrillation is essential, as up to one-third of patients may no longer require a device [58]. When reimplantation is necessary, systems minimizing intravascular hardware, leadless pacemakers or subcutaneous implantable cardioverter-defibrillators, are preferred. Reimplantation should be performed on the contralateral side and postponed until blood cultures are negative, typically [36]:

- ≥72 h after extraction if no vegetations or “ghosts” are present.
- ≥2 weeks if vegetations were observed.

## 7. Predictive Scores for Intra-Hospital Mortality, Surgical Outcomes and 6-Month Mortality

### 7.1. In-Hospital Mortality

In-hospital mortality among patients with IE ranges between 15% and 30% [7,59,60,61]. In-hospital mortality is higher for PVE (19.9%) as compared to CDRIE (15.3%) and NVE (16.2%) [7]. The cases for death were also different being cardiovascular for PVE (mainly heart failure) as compared to non-cardiovascular (mainly sepsis) for both CDRIE and NVE [7].

The latest ESC guidelines on IE describe four major determinants of early prognosis: the infecting microorganism, baseline patient characteristics, non-cardiac complications, and echocardiographic evidence of cardiac structural damage [36]. However, the prognostic impact of several variables remains insufficiently validated, and their relative weight is not quantified, even though some predictors exert a disproportionately higher influence on mortality risk [62].

As a result, several prognostic models have been developed to identify high-risk patients by integrating the most relevant variables into a mathematical weighted algorithm. These models allow stratification into different risk categories. One of the most widely applicable models, particularly in contemporary IE cohorts regarding age distribution, microbiology, and prosthetic valve involvement, is the score proposed by Garcia-Granja et al., specifically developed to predict in-hospital mortality in left-sided IE [62]. The model included PVE, age, heart failure, renal failure, fungal etiology, periannular complications, septic shock, comorbidities, vegetations, left ventricular dysfunction, and SA as independent predictors of death. The score demonstrated excellent discrimination, with an area under the receiver operating characteristic (ROC) curve of 0.85 [62].

Other scoring tools have been proposed for in-hospital mortality prediction in IE, but they often have limited generalizability to the current and heterogeneous IE population. For instance, the EndoPredict-Px score, based exclusively on variables available during the first hours of admission, was validated in a relatively young cohort (median age 56 years), including a high prevalence of rheumatic valve disease (35%) and biological prostheses (45%) [63]. The ASSESS-IE score was validated in an even younger population (mean age 44 years), with a very low proportion of PVE (6%) and high prevalence of streptococcal IE (37%) [64]. Aside of score model, some factors have been shown to be significantly associated to a high in-hospital mortality such as not performing surgery despite indication and vegetation length greater than 10 mm [7].

### 7.2. Postoperative 30-Day Mortality

The risk associated with surgical intervention in patients with IE is considerable and depends on both pre-existing comorbidities and the current clinical condition. Risk assessment should not rely on a single parameter, such as age, but should instead integrate multiple variables [36]. To this end, surgical risk scores have been applied to patients with IE. Two main types of scores exist: general cardiac surgical scores that can also be applied to IE, and specifically developed IE surgical scores [36].

In a recent study evaluating mortality after surgery for IE, the 30-day postoperative mortality rate was reported to be as high as 10.2% [65]. This risk can rise to up to 30% in emergency operations [66]. The mortality after surgery is higher for PVE (39.6%) as compared to CDRIE (34%) and lastly NVE (27.6%).

The EuroSCORE I and II, which are general cardiac surgical risk models applicable also to IE, were shown to overestimate 30-day postoperative mortality [67]. Compared with EuroSCORE I, which overpredicted mortality across all risk levels, EuroSCORE II tended to overpredict risk only when the estimated mortality exceeded 20% [67]. However, EuroSCORE II demonstrated poor performance in emergency surgical settings [67].

Conversely, another study reported that EuroSCORE II performed better than two IE-specific models, named AEPEI II and APORTEI, in predicting 30-day postoperative mortality (area under the ROC curve of 0.751 vs. 0.672 vs. 0.693) [66]. Among its variables, EuroSCORE II includes only one factor directly related to IE (antibiotic therapy at the time of surgery). The remaining parameters reflect the general hemodynamic condition, presence of multiorgan failure, and urgency of surgery. Although EuroSCORE II does not explicitly address local cardiac complications, these may indirectly influence the score through variables reflecting surgical complexity [66].

The Italian group of research for outcome in cardiac surgery validated the EndoScore [65] in a population with IE characterized by a mean age of 59 ± 15 years and a relatively small proportion of patients with multiple valve involvement (20%). Most patients had NVE (79%), predominantly affecting the aortic and mitral valves. Streptococcal infection represented the most common etiology (34.7%), and surgery was performed on a single valve in the majority of cases (79%).

The EndoScore integrated several clinical and operative variables into its model, including age, female sex, left ventricular ejection fraction, chronic obstructive pulmonary disease, preoperative shock, number of valves treated, presence of abscess, and type of microorganism. The discriminative ability of the final model was high, with an area under the ROC curve of 0.836 [65].

### 7.3. Six-Month Mortality Risk

The risk of death in patients with IE, whether managed medically or surgically, remains elevated for up to four months after diagnosis [68]. Both cardiovascular and non-cardiovascular events contribute to this persistently high risk [69,70]. The overall six-month mortality rate averages approximately 25%.

Park et al. proposed a simplified risk score to predict six-month mortality based on four groups of variables: host factors (age and dialysis), IE-related factors (PVE, SA infection, and valve vegetation), complications (NYHA functional class, stroke, paravalvular complications, and persistent bacteremia), and surgical treatment. These parameters are typically available at the time of diagnosis and are independent of surgical management. Using this model, patients can be stratified according to their predicted six-month mortality risk, ranging from 10 percent to 50 percent [60].

## 8. Echocardiography in the Diagnosis of Infective Endocarditis

Echocardiography remains the first-line imaging modality for the diagnosis of IE and represents the cornerstone of Duke-based diagnostic criteria [36,37]. TTE is typically the first-line imaging modality; however, its performance is limited by image quality and prosthetic material, often necessitating TEE to integrate the analysis [36]. Table 3 summarizes the main echocardiographic parameters that should be evaluated in IE patients.

In NVE, TTE shows a wide range of sensitivity (approximately 50–90%) while maintaining consistently high specificity (around 90%), performing best in patients with favorable acoustic windows and larger vegetations [77] (Table 4).

Clinically, this means that a negative TTE cannot reliably exclude IE in patients with high pre-test probability, whereas a positive finding is usually trustworthy given the high specificity. TTE is particularly useful for evaluating right-sided IE, especially when the tricuspid valve is involved, as vegetations are often large. In such cases, particularly in patients with favorable acoustic windows, TTE may be sufficient for diagnosis and for follow-up patients [78,79].

**Figure 1:**
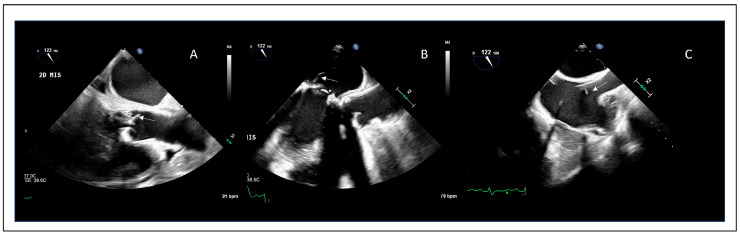
Transesophageal echocardiographic findings across different infective endocarditis setting. (**A**) Native valve infective endocarditis. Mid-esophageal transesophageal echocardiographic view of the native aortic valve showing degenerative calcific changes of the cusps. A thin, filiform echodense structure is visible on the non-coronary cusp (arrow), consistent with a valvular vegetation. (**B**) Prosthetic valve endocarditis. Mid-esophageal transesophageal echocardiographic view of a mitral bioprosthesis demonstrating a filiform echodense formation measuring approximately 10 mm, attached to the prosthetic leaflet (arrow), compatible with prosthetic valve involvement in infective endocarditis. (**C**) Cardiac device-related infective endocarditis. Bicaval transesophageal echocardiographic view showing a filiform echodense structure adherent to an intracardiac pacing lead (arrow), consistent with a vegetation attached to the lead.

TEE provides markedly superior spatial resolution and is therefore recommended when TTE is negative or inconclusive in the presence of persistent clinical suspicion [79] (Figure 1).

In NVE, two-dimensional (2D) TEE achieves very high sensitivity (90–100%) and specificity exceeding 90%, establishing it as the reference standard for echocardiographic diagnosis [80]. The incremental value of three-dimensional (3D) TEE is mainly related to improved morphological assessment of vegetations, more accurate size measurements, and better characterization of features associated with embolic risk, rather than a substantial increase in diagnostic sensitivity alone [75].

The diagnostic advantage of TEE becomes even more relevant in PVE, where acoustic shadowing and reverberation artifacts substantially reduce the sensitivity of TTE (40–70%) [81,82].

In this context, 2D TEE improves sensitivity to approximately 82–96% [81,82], while contemporary 3D TEE further enhances diagnostic performance, particularly for the detection and delineation of periannular complications, with reported sensitivities exceeding 90% [71,81,83]. Periannular complications are more frequently observed in prosthetic valve endocarditis; therefore, in patients with prosthetic material, TEE plays a key role in accurate detection [36].

In CDRIE, TTE has limited diagnostic utility, with sensitivities generally below 50%, reflecting its inability to reliably visualize intracardiac leads and small lead-associated vegetations [82]. TEE is therefore mandatory in this setting, achieving sensitivities of 85–95% [84]. 3D TEE provides additional value by offering improved anatomical definition of lead-tissue interactions and more comprehensive visualization of vegetation extent [84].

Overall, these findings support a stepwise echocardiographic approach to IE diagnosis, in which TTE serves as an initial screening tool, while TEE, particularly when complemented by 3D imaging, represents the definitive technique across NVE, PVE, and CDRIE.

## 9. Diagnostic Performance of TEE Versus Cardiac CT in Infective Endocarditis

When comparing TEE and cardiac CT, the clinical meaning of differences in sensitivity, specificity, predictive values, and diagnostic accuracy becomes evident when stratifying by EI subtypes (NVE vs. PVE) and lesion complexity (vegetations vs. IE intracardiac complications). For vegetation detection, TEE consistently outperformed CT in NVE, with higher sensitivity (91.1% vs. 80.0%) and specificity (87.5% vs. 62.5%), translating into superior diagnostic accuracy (90.6% vs. 77.3%) [73] (Table 5).

Clinically, this indicates that in NVE, TEE is both more reliable for identifying vegetations and more trustworthy when excluding them. Measurements of vegetation size are well correlated between TEE and cardiac CT for large vegetations (>10 mm) (r = 0.6, *p* < 0.01), whereas small vegetations (<10 mm) are frequently underdetected by cardiac CT [71].

In PVE, sensitivities for vegetation detection were identical for TEE and CT (81.8%), reflecting the intrinsic limitations imposed by prosthetic material on both techniques [73]. However, the markedly lower specificity of CT (50.0% vs. 75.0% for TEE) resulted in a substantial drop in diagnostic accuracy (50.0% vs. 80.0%) [73], highlighting that approximately one out of two patients without IE may be falsely classified as positive by CT in this setting, particularly in prosthetic valves, where imaging artifacts and structural postoperative changes are frequent.

For vegetation detection, both TEE and CT showed high positive predictive values (TEE 90–98%, CT 82–92%) [73], indicating that a positive finding is highly reliable in patients at high risk for IE. In contrast, negative predictive values were modest for both techniques, particularly in PVE (TEE = 60%, CT = 50%), indicating that a negative imaging study does not safely exclude disease and supporting the need for repeat or multimodality imaging when clinical suspicion persists.

**Figure 2:**
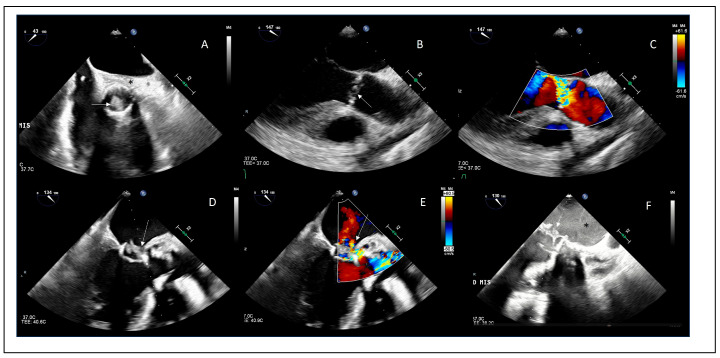
Transesophageal echocardiography findings of intracardiac complications of infective endocarditis. Panel (**A**). Aortic abscess. Two-dimensional transesophageal echocardiography showing a periprosthetic aortic abscess (asterisk) in a patient with a biological aortic valve prosthesis. The arrow indicates a rounded, sessile vegetation adherent to the prosthetic leaflets, consistent with prosthetic valve endocarditis. Panel (**B**). Leaflet perforation. Two-dimensional transesophageal echocardiography demonstrating a perforation of a native aortic valve leaflet secondary to infective endocarditis, indicated by the arrow. Panel (**C**). Leaflet perforation. Corresponding color Doppler imaging confirms severe aortic regurgitation with an eccentric jet, resulting from IE-related leaflet perforation. Panel (**D**). Fistula. Shows two-dimensional transoesophageal echocardiography in a long-axis view, demonstrating a perivalvular abscess secondary to infective endocarditis involving the mitro-aortic continuity. The abscess cavity extends into the fibrous intervalvular region and is complicated by the formation of a fistulous communication between the left ventricular outflow tract (LVOT) and the left atrium (arrow). Panel (**E**). Fistula. Depicts the same view with color Doppler imaging, clearly visualizing turbulent systolic flow across the fistulous tract from the LVOT into the left atrium, confirming the presence of a hemodynamically significant intracardiac fistula (arrow) as a complication of infective endocarditis. Panel (**F**). Severe prosthetic dysfunction. Two-dimensional transoesophageal echocardiography demonstrates severe dysfunction of the mitral bioprosthesis secondary to a large vegetation adherent to the atrial side of a prosthetic mitral leaflet (arrow). The vegetation results in marked obstruction of transprosthetic flow, leading to severe bioprosthetic mitral stenosis. Prominent spontaneous echo contrast (“smoke effect”) is evident shown by the asterisk within the left atrium (asterisk), reflecting severe blood flow stasis due to critical valve obstruction.

The complementary role of cardiac CT is most evident in the assessment of periannular complications. For abscess detection, CT showed higher sensitivity than TEE (77.3% vs. 72.7%) [73], whereas TEE maintained higher specificity [73] (Figure 2).

This means that CT is more effective at identifying periannular abscesses when they are present, particularly when infection extends beyond the valve plane, whereas the higher specificity of TEE makes it more reliable in confirming true abscesses and reducing false-positive diagnoses.

This difference was even more pronounced for pseudoaneurysms, where CT achieved 100% sensitivity, while maintaining excellent specificity and diagnostic accuracy [73]. These data support a tailored multimodality imaging strategy, in which TEE remains the cornerstone for vegetation detection, particularly in native valves, and CT provides critical incremental value for periannular complications. The diagnostic accuracy of cardiac CT may be limited by motion artifacts and the requirement for optimal heart rate control during image acquisition. Conversely, it provides valuable additional information on coronary anatomy, which is particularly useful in patients undergoing cardiac surgery [85]. Cardiac CT does not provide information on valvular function or hemodynamic consequences and exposes patients to ionizing radiation. In addition, the use of iodinated contrast agents may limit its applicability in patients with renal impairment [85].

## 10. Nuclear Medicine Imaging in Infective Endocarditis

### 10.1. Diagnostic Accuracy of FDG-PET/CT in IE

FDG is a glucose analogue avidly taken up by cells with high metabolic activity, including activated inflammatory cells, allowing rapid acquisition and high spatial resolution when combined with PET/CT technology [36,37].

FDG-PET/CT is recommended in cases of suspected NVE, PVE, and CDRIE especially when conventional echocardiography is inconclusive or limited by artifact, and for the detection of systemic or pulmonary septic embolization (excluding central nervous system involvement when contrast-enhanced CT is not performed). In selected patients, it may also be used to assess treatment response, particularly during prolonged antimicrobial therapy or in PVE when surgery is contraindicated [36,37].

Contemporary diagnostic frameworks, including the most recent European and Duke-ISCVID criteria, explicitly incorporate abnormal FDG uptake as a major diagnostic criterion in selected settings, reflecting the growing recognition of nuclear imaging as an essential component of a multimodality diagnostic strategy [36,37].

A recent meta-analysis evaluated the diagnostic accuracy of FDG-PET/CT for IE, reporting markedly different performance across disease subtypes [86] (Table 6).

Sensitivity was low in NVE (31%), substantially higher in PVE (86%), and intermediate in CDRIE (72%), while specificity remained high across all settings (98%, 84%, and 83%, respectively) [86].

In NVE, the combination of very low sensitivity (31%) and extremely high specificity (98%) indicates that FDG-PET/CT has limited value as a rule-out test [86]. Clinically, a positive scan strongly supports the presence of infection, whereas a negative result is frequent even in confirmed cases and therefore cannot be used to exclude NVE. This pattern likely reflects the limited metabolic detectability of small, highly mobile native valve vegetations.

In fact, FDG-PET/TAC has limited sensitivity for the detection of isolated vegetations, particularly when vegetations are small and associated with low metabolic activity [87].

In contrast, in PVE, FDG-PET/CT demonstrated both high sensitivity (86%) and good specificity (84%) [86]. From a clinical standpoint, a positive scan provides strong diagnostic confirmation, while a negative scan substantially reduces the likelihood of PVE, making FDG-PET/CT particularly valuable as a complementary modality when echocardiography is inconclusive.

**Figure 3:**
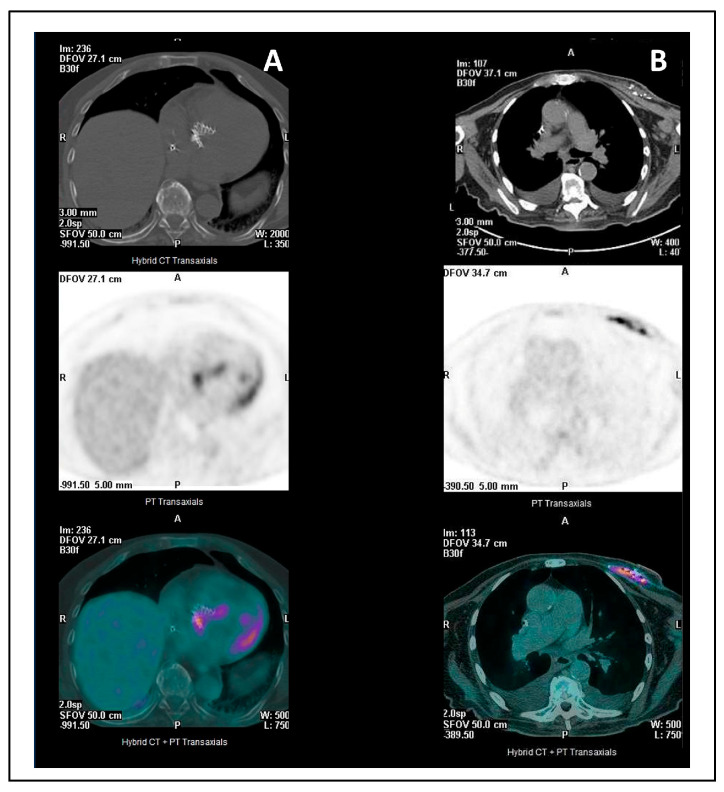
FDG-PET/CT findings in prosthetic valve endocarditis and cardiac device-related infective endocarditis. Panel (**A**). Prosthetic valve endocarditis with perivalvular abscess. From top to bottom: Non-contrast-enhanced CT (low-dose CT for attenuation correction and anatomical localization) showing a biological aortic valve prosthesis with a perivalvular low-attenuation collection consistent with abscess formation. Attenuation-corrected PET demonstrating focal increased FDG uptake in the periprosthetic region. Fused PET/CT images confirming intense, focal FDG uptake spatially corresponding to the perivalvular collection, consistent with metabolically active infection. Panel (**B**). Cardiac implantable electronic device pocket infection. From top to bottom: Non-contrast-enhanced CT showing soft tissue thickening at the generator pocket site. Attenuation-corrected PET demonstrating focal FDG uptake at the device pocket. Fused PET/CT images confirming localized increased FDG uptake at the pacemaker pocket, consistent with active device-related infection. These examples highlight the complementary value of anatomical CT and metabolic PET imaging for the detection of intracardiac and extracardiac infectious complications, particularly in prosthetic valve endocarditis and cardiac device-related infective endocarditis.

Similarly, in CDRIE, the combination of moderate-to-high sensitivity (72%) and good specificity (83%) supports the use of FDG-PET/CT to confirm device-related infection, especially in patients with systemic signs or persistent bacteremia [86] (Figure 3).

However, the residual false-negative rate underscores that a negative scan cannot definitively exclude infection in high-risk patients and should be interpreted within a multimodality diagnostic framework.

The major strength of FDG-PET/CT lies in its integration with echocardiography. Concordant findings between the two modalities, particularly when both localize infection to the same cardiac structure, markedly enhance diagnostic confidence, with reported combined sensitivity of 96% and specificity 100% [88].

The presence of extracardiac FDG uptake consistent with septic emboli, particularly involving the spleen, lungs, or osteoarticular system, significantly strengthens the diagnostic suspicion of infective endocarditis [89].

FDG-PET/CT image interpretation may be affected by several confounding factors that can result in both false-positive and false-negative findings. Post-surgical inflammation, particularly within the first three months after surgery, metabolically active atherosclerotic plaques, recent non-infected thrombi, and even malignant tumors may all show increased FDG uptake and mimic infection [86,90] (Table 7).

Current European Society of Cardiology and International Society for Cardiovascular Infectious Diseases guidelines strongly recommend performing FDG-PET/CT as early as possible, ideally before the initiation of antibiotic therapy or, at the latest, within the first week of treatment [36,37]. FDG-PET/CT performed after several weeks of antibiotic therapy has a markedly reduced negative predictive value and therefore cannot reliably exclude ongoing infection [93]. In patients who have recently undergone valve surgery, FDG-PET/CT is preferably deferred for at least three months to reduce the risk of false-positive findings related to sterile postoperative inflammation. However, in the presence of urgent clinical suspicion of PVE, such as persistent fever or positive blood cultures during hospitalization, earlier imaging may be justified, provided that results are interpreted in close clinical correlation [89,94]. Libman-Sacks endocarditis, a non-bacterial inflammatory valvular disease associated with systemic lupus erythematosus, represents a notable exception, as it typically shows absent or minimal FDG uptake, a feature that may be crucial in excluding active IE [90].

### 10.2. Patient Preparation and Image Interpretation: Visual and Semiquantitative Approaches

Appropriate patient preparation is critical to ensure diagnostic accuracy, as physiological myocardial glucose uptake remains one of the main confounders in cardiac FDG-PET/CT [95]. Current guideline recommendations are summarized in Table 8 [95].

Image analysis is performed at two complementary levels: visual assessment and semiquantitative analysis. Visual interpretation focuses on the pattern of FDG uptake within myocardial structures or along intracardiac devices. Uptake patterns are classified as focal or diffuse, with diffuse uptake further categorized as homogeneous or heterogeneous [96]. Imaging findings considered suggestive of IE typically consist of focal or heterogeneous FDG uptake on attenuation-corrected images that remains clearly identifiable on non-attenuation-corrected images, helping to exclude attenuation artifacts [95].

Confirmation on non-attenuation-corrected images is particularly important in PVE and CDRIE, as metallic components may generate attenuation artifacts that falsely appear as increased FDG uptake on attenuation-corrected images [97].

Semiquantitative analysis is based on standardized uptake value (SUV) measured on attenuation-corrected image and on its ratio with reference regions to correct for background activity.

The maximal SUV (SUV max) measurement is typically obtained using an isocontour-based volume of interest defined by a threshold of 40% of the maximal SUV. It includes both the target lesion and adjacent blood pool activity [88]. The SUV ratio is calculated as the ratio between lesion SUV max and the mean SUV of the blood pool in the descending aorta [88]. A major limitation of this approach is the lack of a universal cut-off value, as optimal thresholds may vary according to scanner characteristics, image reconstruction protocols, and local standardization procedures.

Current guidelines suggest a SUV cut-off value greater than 3.3, however this threshold is derived from limited evidence and requires further validation in larger and prospective studies [95]. From a research perspective, several authors have investigated specific SUV cut-off values, however, these thresholds are often derived from highly controlled research protocols and may not be readily generalized to routine clinical practice (Table 9).

### 10.3. Radiolabeled Leukocyte Scintigraphy for Assessing IE

Autologous radiolabeled leukocyte scintigraphy is based on labeling circulating leukocytes to visualize sites of active infection and represents the reference standard for its very high specificity in differentiating septic processes from sterile inflammation. This technique is particularly useful in cases of equivocal or inconclusive FDG-PET/CT findings [99].

Radiolabeled leukocyte scintigraphy requires patient fasting and, when clinically feasible, temporary discontinuation of antibiotic therapy for 10–14 days in order to improve sensitivity. Image acquisition is performed at multiple time points (e.g., 2–4 h and 20–24 h after tracer injection), and single-photon emission computed tomography combined with CT (SPECT/CT) is mandatory to ensure accurate anatomical localization of tracer uptake [99].

The criterion for active infection is a progressive increase in the intensity or extent of tracer uptake on delayed images compared with early acquisitions, occurring at the valvular plane in NVE and PVE or at the device pocket or leads in CDRIE. In contrast, decreasing tracer uptake over time is more suggestive of sterile inflammation [99]. Whole-body leukocyte SPECT/CT provides higher specificity than FDG-PET/CT, reaching values close to 100%, but at the cost of lower sensitivity (approximately 60%). For this reason, it is particularly valuable when FDG-PET/CT results are non-diagnostic or equivocal, despite higher costs and more limited availability [100]. A major limitation of leukocyte scintigraphy is the risk of false-negative findings, which may occur in the presence of small infectious foci (vegetations < 10 mm) or in infections caused by microorganisms such as *Candida* spp., *Enterococcus* spp., or *Staphylococcus epidermidis.* These pathogens may evade leukocyte recruitment through biofilm formation, thereby rendering the infection metabolically occult on leukocyte imaging [101].

## 11. Management of Infective Endocarditis: The Evolving Roles of OPAT and Oral Step-Down Therapy

### 11.1. Outpatient Parenteral or Oral Antibiotic Therapy

The management of IE has undergone substantial evolution in recent years, driven by the need to balance effective antimicrobial therapy, patient safety, and healthcare resource optimization. Traditionally, IE required prolonged in-hospital IV antibiotic therapy lasting 4–6 weeks, primarily to ensure bacteremia clearance, monitor for complications, and manage potential hemodynamic deterioration. However, extended hospitalization is associated with well-recognized drawbacks, including increased exposure to nosocomial infections, antimicrobial resistance, financial burden, and treatment-related morbidity. These limitations underscore the need for safe strategies that allow earlier transition to outpatient care. Two complementary approaches have therefore gained prominence: outpatient parenteral antibiotic therapy (OPAT) and oral step-down antibiotic therapy following initial IV treatment.

### 11.2. OPAT: From Restrictive Criteria to OPAT-GAMES Patient Selection

OPAT consists of administering IV antibiotics outside the hospital setting, typically using the same regimen prescribed during hospitalization, and is supported by two physician visits per week along with a daily visit from a nurse [102]. OPAT can be performed both in a long-term care structure and at the patient home [103]. It has long been recognized as an attractive alternative to prolonged hospitalization, offering comparable efficacy, greater patient comfort, and reduced healthcare costs when applied to carefully selected candidates [36]. Early recommendations, such as the 2001 Infectious Diseases Society of America (IDSA) criteria, were intentionally conservative [104]. They limited OPAT to highly stable patients with non-aortic native-valve IE caused by low-virulence organisms (mainly viridans-group streptococci), and excluded those with hemodynamic instability, persistent bacteremia, prosthetic-valve involvement, perianular complications, or surgical indications [104]. Over time, it became evident that these narrow criteria excluded the majority of IE patients and did not reflect contemporary microbiological or clinical realities [102].

In response, the OPAT-GAMES criteria, developed by the Spanish GAMES network, introduced a broader and more pragmatic framework. Rather than focusing solely on microbiology or valve type, OPAT-GAMES assess patient eligibility based on resolution of the acute phase and clinical stabilization [102,103].

The acute phase may be considered resolved after more than 3 weeks of hospital-based IV antibiotic therapy for NVE and PVIE, and after more than 2 weeks for CDRIE [36]. In cases caused by oral streptococci or *Sreptococcus gallolyticus*, OPAT can be initiated earlier, after more than 10 days for NVE or PVE and more than 7 days for CDRIE [36].

The exclusion criteria of the OPAT-GAMES model include Child–Pugh B/C liver cirrhosis, severe central nervous system emboli, undrained abscesses, perianular extension requiring surgery, active IV drug use, and highly difficult-to-treat microorganisms (for example, MRSA requiring prolonged inpatient monitoring or multidrug-resistant gram-negative bacteria). Additional exclusions apply to pathogens requiring complex antimicrobial regimens or associated with a high risk of treatment-related toxicity [103].

Under these expanded criteria, more than half of IE patients in large multicenter cohorts were eligible for OPAT, compared with approximately 20% under the IDSA criteria [102]. In the GAMES prospective cohort, encompassing 2000 consecutive IE cases across 25 Spanish hospitals, OPAT proved both safe and effective. Mortality (8% vs. 7.4%, *p* = 0.103) and recurrence rates (3.1% vs. 2.5%, *p* = 0.546) were similar between OPAT and hospital-based antibiotic therapy (HBAT) [103]. Readmission rates were slightly higher among OPAT patients (approximately 2% absolute increase), but these were primarily related to antibiotic toxicity, wound complications after cardiac surgery, or catheter-related issues [103].

Importantly, patients who underwent OPAT despite not meeting OPAT-GAMES criteria had substantially higher readmission rates (23.8% vs. 16.4%), emphasizing the critical importance of strict patient selection [103].

Beyond these clinical outcomes, OPAT offers significant advantages in terms of safety and cost. Prolonged hospitalization increases the risk of antimicrobial-resistant infections, adverse drug events, venous thromboembolism, functional decline, and healthcare-associated infections, all of which can be mitigated by appropriate outpatient management. From an economic perspective, IE imposes substantial financial strain. A Spanish study estimated the average treatment-related cost of IE at approximately €15,000 per patient in 2014, with hospital stay representing one of the major cost drivers [105].

Patients treated with OPAT who met the OPAT-GAMES eligibility criteria experienced a significant reduction in-hospital length of stay, with a median decrease of 19 days (interquartile range 13–29) [103]. This represents a meaningful improvement in healthcare resource utilization. Consequently, the ability to safely shorten inpatient duration through OPAT carries substantial economic implications.

Patients receiving OPAT experienced an improved psychological well-being. Better sleep quality, and early mobilization compared with those undergoing prolonged hospitalization [106]. These factors may contribute to a reduced risk of venous thromboembolic events and are particularly beneficial in elderly patients [107].

However, OPAT also has limitations. Reduced direct clinical observation may delay the recognition of clinical deterioration, particularly in patients living alone or with multiple comorbidities. Adequate social and caregiver support should be ensured, and patients must be educated to recognize early warning signs [106]. Therefore, careful patient selection, including assessment of social support, is essential before initiating OPAT [108].

### 11.3. Transition to Oral Therapy: Evidence from the POET Trial and Real-World Validation

In parallel with OPAT, compelling evidence now supports the use of oral step-down antibiotic therapy for IE following an initial phase of IV treatment. The pivotal POET trial fundamentally shifted this paradigm by demonstrating that a carefully selected, clinically stabilized, and uncomplicated cohort of patients with left-sided IE involving either NVE or PVE could safely transition to oral therapy after receiving at least 10 days of IV antibiotics [109]. Oral step-down therapy requires the administration of at least two oral antibiotics, selected on the basis of known bacterial resistance patterns and national guideline recommendations for antimicrobial treatment [110]. Clinical stabilization was strictly defined and included the absence of abscess formation on echocardiography. The full set of POET inclusion criteria is summarized in Table 10 [109].

The population enrolled in POET had specific clinical characteristics: streptococci were the most frequently isolated pathogens (45.8%); only 26.9% of patients had PVE; and the infection more often involved the aortic valve (54%) than the mitral valve (35%). In addition, only 4% of patients had vegetations larger than 9 mm or moderate-to-severe valvular regurgitation (10% of patients) [109]. Patients with impaired immune function, such as those receiving prednisolone > 10 mg/day or equivalent, were excluded [109].

All participants underwent TEE at least twice: once before randomization to exclude local complications (such as abscess or fistula), and again at the completion of antibiotic therapy (whether oral or intravenous) to ensure an adequate therapeutic response [109].

POET demonstrated the non-inferiority of oral therapy compared with IV treatment for the composite primary outcome (all-cause mortality, unplanned cardiac surgery, embolic events, or relapse of bacteremia) at 6 months [109]. Importantly, long-term follow-up at 5 years revealed a significantly lower occurrence of the composite endpoint in the oral-therapy group, driven primarily by a reduction in all-cause mortality, suggesting not only safety but also potential long-term clinical benefit [111].

Real-world data further confirmed the safety of this approach: one observational study showed that the POET strategy yielded similar 90-day mortality and readmission rates compared with continued IV antibiotic therapy [112].

The POETry study applied POET criteria in a cohort of 562 IE patients. Participants were randomized to an oral step-down antibiotic regimen versus traditional IV therapy [110]. The study showed that the oral strategy was associated with a significant reduction in-hospital length of stay (24 days vs. 43 days, *p* < 0.001). A reduction in all-cause mortality was also observed in the oral group (5% vs. 16%, *p* = 0.005) [110]. However, it should be noted that the group receiving IV therapy had a worse baseline clinical and microbiological profile, with a higher prevalence of SA infection and paravalvular abscess [110].

## 12. The Role of the Multidisciplinary Team and Its Impact on the Management of IE

The management of IE has become increasingly complex due to evolving patient characteristics, a rising prevalence of virulent pathogens such as SA and Enterococcus spp., greater comorbidity burden, and the growing use of intravascular and cardiac implantable devices. These shifts, combined with advances in diagnostic imaging and therapeutic options, have underscored the need for coordinated, expert-driven care strategies. Despite these changes, high-quality randomized trials remain scarce, and much of the contemporary evidence guiding IE diagnosis and management originates from expert consensus or analyses of large registries [113]. Consequently, a multidisciplinary team (MDT) approach has emerged as a recommended standard of care for IE, aimed at optimizing diagnostic accuracy, streamlining clinical decision-making, and improving patient outcomes.

Early experiences demonstrated that structured multidisciplinary oversight can substantially standardize and improve IE management. For example, one center developed a dedicated MDT encompassing specialists from microbiology, cardiology, infectious diseases, cardiac surgery, and other relevant fields [114]. This group designed a unified protocol that included standardized use of antimicrobial agents, defined treatment duration, consistent indications for surgery, and systematic follow-up [114].

The implementation of this coordinated strategy resulted in a significant reduction in 1-year mortality (HR 0.41, *p* = 0.008), and multivariate analysis demonstrated that the MDT was a strong protective factor against adverse events (HR 0.26, *p* = 0.01) [114].

The composition and function of MDTs vary across institutions, reflecting local resources, epidemiological patterns, regional variability, and socioeconomic factors [113].

Contemporary guidelines propose a tiered model in which hospitals are classified into primary, secondary, and tertiary levels according to their diagnostic and therapeutic capabilities. Primary-level facilities manage uncomplicated cases without immediate surgical indication, while secondary-level hospitals function as “functional” reference centers equipped with an MDT. These teams oversee patients with favorable clinical profiles or those at increased risk of complications, provided that no urgent surgical intervention is required.

Functional reference centers are required to include an echocardiography laboratory capable of performing TEE, a coronary care unit, infectious disease specialists, a microbiology laboratory, and direct access to advanced imaging modalities such as FDG-PET/CT and cardiac magnetic resonance. Cardiac surgery is not required to be physically available on site; however, rapid transfer to a structural reference center must be guaranteed, ideally within 24–48 h in urgent cases. Cardiac surgical consultation should be accessible within 48 h for non-urgent cases, and cardiac surgeons should participate in the weekly meetings of the MDT through remote review of echocardiography and other imaging studies via shared digital platforms [115].

Tertiary-level facilities serve as “structural” reference centers with cardiac surgery on site and are responsible for managing complex presentations including heart failure unresponsive to medical therapy.

Indications for rapid patient transfer to a tertiary center with cardiac surgery on site include the need for emergency surgery, aortic or mitral valve dysfunction causing pulmonary oedema, and uncontrolled infection.

Most MDTs include a core group of cardiologists (often with imaging expertise), infectious disease physicians, and cardiac surgeons, with more recent incorporation of specialists in neurology, nuclear medicine, internal medicine, and clinical pharmacy [113]. This expansion reflects the increasing clinical complexity of IE and the growing challenges associated with imaging interpretation [113]. Many teams also integrate a clinical nurse specialist to facilitate communication, coordinate investigations, and support follow-up [113]. MDTs typically meet at least weekly, but urgent evaluations occur whenever clinical deterioration or acute surgical needs arise [113]. To ensure timely surgical referral, the team must include or have immediate access to a cardiovascular surgeon capable of performing advanced valve and aortic procedures that cannot be deferred [113].

The primary goal of IE MDTs is to expedite and improve the diagnosis and treatment of suspected or confirmed IE [113]. Their role spans the entire disease trajectory, including initial assessment, verification of diagnostic probability (definite, possible, or indeterminate IE), and guidance on appropriate imaging. Advanced imaging modalities, such as FDG-PET/CT, are increasingly integrated into MDT workflows to evaluate prosthetic valve involvement, detect extracardiac complications, and assess the pattern and intensity of FDG uptake when IE probability remains intermediate. MDTs also help address the broad variability in length of stay and patterns of care observed across different institutions.

Multiple studies have demonstrated the clinical value of MDT implementation. Approximately 25–50 percent of IE patients ultimately undergo surgical management [116], and MDTs ensure timely identification of surgical indications and expedited surgical intervention when required. A majority of institutions adopting MDTs reported improved adherence to guideline-directed therapy, greater rates of appropriate antimicrobial selection, and more consistent use of recommended diagnostic evaluations [117]. Importantly, a meta-analysis evaluating MDT implementation revealed a significant reduction in short-term mortality, with an overall risk ratio of 0.61, highlighting the tangible survival benefits associated with coordinated multidisciplinary care [117]. Short-term mortality reductions were documented both during hospitalization and up to 30 days after diagnosis [117].

Observational findings also indicate a decreased hospital length of stay, as well as a reduced time from diagnosis to surgery [118,119].

Beyond direct clinical management, MDTs play a crucial role in patient and provider education [113]. Education focuses on early recognition of IE symptoms, oral hygiene, and antibiotic prophylaxis when indicated, and long-term health maintenance practices. Since the risk of recurrence or relapse is highest within the first year following an IE episode [120,121], clear communication and structured follow-up are essential. MDTs ensure that patients are well-informed about their condition, the rationale for therapeutic choices, and the significance of adherence to antimicrobial therapy, follow-up imaging, and preventive measures.

In addition to improving clinical outcomes, MDTs also play an important role in enhancing patient and caregiver satisfaction with medical decisions [122]. Given the multidisciplinary nature of IE, an MDT approach helps ensure consistent communication and alignment of clinical decisions among specialists. This reduces the risk of conflicting opinions that may negatively impact on patient confidence.

Establishing an effective MDT requires institutional support and motivated clinicians. Teams must invest time in developing management algorithms, referral pathways, and communication platforms [113]. Some centers dedicated their initial months to designing comprehensive protocols addressing laboratory and imaging evaluation, timing of advanced imaging, screening for substance use disorders, engagement with addiction treatment services, neurologic assessment strategies, and indications for medical versus surgical management [123]. Outreach efforts, such as electronic or paper communication with referring centers, local hospitals, and remote communities, can broaden the reach of MDTs and enhance early referral and diagnosis. The present review addresses several unmet needs in the management of infective endocarditis, highlighting their clinical context and potential impact on patient outcomes, and proposes a multidisciplinary approach to address these challenges, as summarized in Table 11.

## 13. Conclusions

IE is a complex disease characterized by sophisticated pathophysiological mechanisms, in which highly virulent pathogens have evolved multiple strategies to evade host immune defenses. Its diagnosis requires an integrated approach combining microbiological investigations with advanced imaging techniques. Nevertheless, clinical suspicion and bedside findings remain central to the diagnostic process, as results from individual tests may be influenced by confounding factors and therefore require careful contextual interpretation.

Current diagnostic strategies for IE rely on a stepwise multimodality imaging approach guided by clinical probability. TTE remains the first-line test in patients with low pre-test probability, while TEE is required in cases of intermediate or high suspicion to improve sensitivity and identify intracardiac complications. Cardiac CT provides complementary anatomical information, particularly for perivalvular extension and prosthetic valve assessment. FDG-PET/CT adds metabolic insights and plays a pivotal role in PVE and CDRIE, as well as in the detection of extracardiac septic emboli. The integrated use of these techniques improves diagnostic accuracy when interpreted within the appropriate clinical context. The MDT model has demonstrated a clear potential to improve the quality, consistency, and outcomes of patient care. By facilitating timely diagnosis, appropriate antimicrobial therapy, early access to cardiac surgery when indicated, and structured long-term follow-up, the MDT approach contributes to reduced mortality and greater standardization of care pathways in a condition historically associated with high variability and poor outcomes.

## 14. Future Directions

Future efforts should focus on the development of structured intra-hospital and regional diagnostic and therapeutic care pathways that define shared protocols for infective endocarditis prevention, diagnosis, treatment, and follow-up. These pathways should also include clear criteria for patient transfer and back-transfer between tertiary referral centers and non-tertiary hospitals, ensuring continuity of care across different healthcare settings.

The implementation of OPAT programs, and when appropriate an early switch to oral antibiotic therapy, represents an additional key strategy. These approaches require close coordination with outpatient services and primary care providers to ensure safety, adherence, and monitoring. When appropriately applied, OPAT-based strategies have the potential to reduce hospitalization duration, optimize resource utilization, and lessen the overall burden of IE on healthcare systems.

## Figures and Tables

**Table 1:** Comparison of major and minor diagnostic criteria for IE across classifications.

Classification	Major Microbiological Criteria	Major Imaging/Pathological Criteria	Minor Criteria (Clinical and Predisposition)	Main Characteristics
Original Duke (1994)	Typical microorganisms (including enterococci acquired in the community, in the absence of a primary focus). No standardized serologic definitions	Echocardiographic evidence of vegetation, abscess, or new prosthetic valve dehiscence (no distinction between TEE and TTE)	Predisposing heart condition or IV drug use; fever ≥ 38 °C; vascular and immunologic phenomena	First standardized diagnostic framework. Low sensitivity in prosthetic valve and device-related infections
Modified Duke (2000)	Formalized definition of persistent bacteraemia; inclusion of *Coxiella burnetii* as a major criterion based on serology; exclusion of single positive cultures for CoNS and rare IE pathogens	Improved echocardiographic definitions; greater role of TEE	Refinement of minor criteria; removal of echocardiographic findings from minor criteria	Improved sensitivity vs. original Duke, but still limited for prosthetic material
ESC 2015	Modified Duke microbiology	FDG-PET/CT (recommended ≥3 months after implantation to reduce false positives) or cardiac CT abnormalities as major criteria in PVE	Minor criteria derived from modified Duke classification	Formal inclusion of nuclear imaging. Reduced specificity in high-risk populations
ESC 2023	Similar to ESC 2015; Enterococcus faecalis considered a typical microorganism regardless of acquisition setting	Expanded multimodality imaging (FDG-PET/CT, cardiac CT)	Expanded vascular phenomena, including spondylodiscitis and silent embolic events detected by brain MRI or whole-body CT. Prosthetic valve and intracardiac devices considered predisposing conditions	Higher sensitivity across settings, but lower specificity, especially in CDRIE patients
Duke-ISCVID 2023	Expanded list of typical microorganisms; additional pathogens typical in presence of intracardiac prosthetic material	Pathological evidence from infected removed tissue; surgical inspection; expanded role of FDG-PET/CT in NVE and PVE	Expanded predisposing conditions (e.g., bicuspid aortic valve, previous IE, hypertrophic cardiomyopathy, more than mild valvular regurgitation); refined rejection criteria (e.g., rapid resolution of infection, non-bacterial thrombotic endocarditis).	Highest sensitivity, especially in PVE and CDRIE. Risk of overclassification in lead-related infections

Notes: TEE = transesophageal echocardiography; FDG-PET/CT = fluorodeoxyglucose positron emission tomography/computed tomography; IV = intravenous; PVE = prosthetic valve endocarditis; CDRIE = cardiac device-related infective endocarditis; IE = infective endocarditis; CoNS = coagulase-negative staphylococci; MRI = magnetic resonance imaging; NVE = native valve infective endocarditis.

**Table 2:** Preventive strategies for CDRIE.

Avoiding heparin bridging in patients on vitamin K antagonist or DOACs.
Continuing warfarin in selected patients when appropriate.
Interrupting DOACs without bridging, according to renal function.
Reducing antiplatelet burden, particularly avoiding perioperative DAPT when clinically feasible.
Ensuring rigorous hemostasis and proper compression post-procedure

Notes. DOAC = direct oral anticoagulant; DAPT = dual antiplatelet therapy; CDRIE = cardiac device-related infective endocarditis.

**Table 3:** Echocardiographic findings in IE and corresponding definitions.

Finding	Echocardiographic-Based Description
Vegetation (definition)	An oscillating echogenic mass (also non-oscillating mass can occur) attached to a valve, endocardial surface, prosthetic material, or intracardiac device [71]
Vegetation (presence)	Present: clearly identifiable mass with independent motion. Absent: no mass detected. Possible (not clearly excludable): suspected mass that cannot be confidently excluded as a vegetation due to marked valvular thickening, artifacts, or limited spatial resolution [72]
Valve erosion	Endocardial tissue discontinuity confirmed by color Doppler [73]
Site of attachment of the vegetation	Valve leaflet (mitral, aortic, tricuspid, pulmonary; atrial vs. ventricular side), annulus, prosthesis, intracardiac lead, or intravascular catheter [74]
Number of vegetations	Single vs. multiple (2 or more vegetations) [74,75]
Maximal length (major axis)	Measure the maximum length, with optional use of 3D cropping to obtain the true 2D plane; categorical reporting may classify vegetations as ≤10 mm or >10 mm [75]
Maximal width (minor axis)	Measure orthogonally to the major axis [74], with optional use of orthogonal planes on MPR [76]
Vegetation area	Maximal width multiplied by maximal length [74] or direct area measurement [76]
Morphology (shape and attachment)	Filiform when a narrow attachment with a stalk is present (minor-to-major diameter ratio ≤ 0.5), sessile when a broad implantation base is observed, and grape-like when the morphology consisted of an irregular mass with multiple surface protrusions arising from a common large base [72,76]
Mobility (graded)	Grade 1 (poorly mobile): no independent motion. Grade 2 (moderately mobile): evident independent motion without wide excursion. Grade 3 (highly mobile): marked independent motion with large excursion, typically >90° relative to the attachment point [76] or exceeding the valve coaptation plane [74]
Echogenicity	Echogenicity is graded by comparison with myocardium (grade 1, low) and pericardium (grade 3, high). Grade 2 represents intermediate echogenicity [76]
IE intracardiac complications	
Valve perforation	Focal disruption of leaflet continuity resulting in an abnormal transvalvular flow jet, best visualized with color Doppler imaging [71]
Valve aneurysm	Localized saccular deformation of a valvular leaflet [71]
Paravalvular abscess	Circumscribed nonhomogeneous region (alternatively echolucent or echodense mass) with irregular border within the perivalvular tissue, lacking color Doppler flow signals [71]
Pseudoaneurysm	Perivalvular cavity communicating with the cardiac lumen, characterized by pulsatile expansion and detectable flow on color Doppler interrogation [71]
Intracardiac fistula	Abnormal communication between cardiac chambers or vascular structures, demonstrated by continuous or systolic–diastolic flow on color Doppler [71]
Prosthetic valve dehiscence	Partial separation of a prosthetic valve from the surrounding annular tissue associated with paravalvular regurgitation (with or without abnormal rocking motion) [71]

Notes: 2D = two-dimensional; 3D = three-dimensional; MPR = multiplanar reconstruction.

**Table 4:** Sensitivity and specificity across echocardiography modalities.

Clinical Setting	Modality	Sensitivity (%)	Specificity (%)
NVE	TTE	50–90	~90
	TEE (2D/3D)	90–100	>90
PVE	TTE	40–70	85–90
	TEE 2D/3D	82–96	~94
CDRIE	TTE	<50	NA
	TEE (2D/3D)	85–95	~90

Notes. NVE = native valve infective endocarditis; PVE = prosthetic valve endocarditis; TTE = transthoracic echocardiography; TEE = transesophageal echocardiography; 2D = two-dimensional; 3D = three-dimensional. CDRIE = cardiac device-related infective endocarditis.

**Table 5:** Key diagnostic performance of TEE versus cardiac CT in infective endocarditis.

Target Lesion	Valve Status	Modality	Sensitivity (%)	Specificity (%)	Diagnostic Accuracy (%)
Vegetation	(NVE)	TEE	91.1	87.5	90.6
Vegetation	(NVE)	CT	80.0	62.5	77.3
Vegetation	(PVE)	TEE	81.8	75.0	80.0
Vegetation	(PVE)	CT	81.8	50.0	50.0
Abscess	All patients	TEE	72.7	89.1	83.8
Abscess	All patients	CT	77.3	80.4	79.4
Pseudoaneurysm	All patients	TEE	66.7	100	97.0
Pseudoaneurysm	All patients	CT	100	96.8	97.1

Notes. NVE = native valve infective endocarditis; PVE = prosthetic valve endocarditis; CT = computed tomography; TEE = transesophageal echocardiography.

**Table 6:** Diagnostic performance of FDG-PET/CT across different types of IE.

Accuracy Test	NVE	PVE	CDRIE
Sensitivity	31	86	72
Specificity	98	84	83

Notes. NVE = native valve infective endocarditis; PVE = prosthetic valve endocarditis; CDRIE = cardiac device-related infective endocarditis; FDG-PET/CT = ^18^F-fluorodeoxyglucose positron emission tomography combined with computed tomography; IE = infective endocarditis.

**Table 7:** The most common causes of FDG-PET/CT misinterpretation and their potential impact on diagnostic accuracy.

Condition	False-Positive	False-Negative	Interpretation
Inadequate myocardial FDG suppression [91]	Focal uptake in the basal anteroseptum	**-**	Related to insufficient patient preparation, leading to residual physiological myocardial uptake
Prosthetic heart valve [91]	Mild-to-moderate, symmetrical perivalvular uptake	**-**	Sterile inflammatory response due to foreign body reaction, particularly in the early post-implantation period
Atrial fibrillation [91]	Increased FDG uptake in atrial myocardium	**-**	Reflects increased metabolic demand of atrial myocytes rather than infection
Lipomatous hypertrophy of the interatrial septum [91]	Moderate-to- severe FDG uptake	**-**	Fatty tissue may show increased FDG uptake and mimic pathological uptake near the aortic valve
Surgical adhesive [88]	Intense and persistent focal FDG uptake	-	The use of surgical adhesive has been identified as an independent predictor of false-positive results
Ongoing antibiotic therapy [91,92]	**-**	Reduced or absent FDG uptake at the site of infection	FDG uptake reflects inflammatory cell activity rather than direct pathogen burden; prolonged therapy may suppress metabolic signal
Valve vegetation [91]	**-**	Missed or faint uptake	Limited spatial resolution and lower inflammatory activity compared with perivalvular complications, resulting in reduced sensitivity in NVE
Low inflammatory status (e.g., related to prolonged antibiotic therapy) [88]	**-**	Absent or reduced FDG uptake at the site of infection	A C-reactive protein value < 4 mg/dL has been identified as an independent predictor of false-negative findings

Notes. FDG = fluorodeoxyglucose; NVE = native valve infective endocarditis; FDG-PET/CT = ^18^F-fluorodeoxyglucose positron emission tomography combined with computed tomography.

**Table 8:** Patient preparation and lesion-to-background image analysis.

Step	Recommendation	Rationale
**Dietary preparation** [95]	High-fat, low-carbohydrate diet for 12–24 h before the examination	Suppresses physiological myocardial glucose uptake and improves detection of pathological FDG uptake
**Fasting** [95]	12–18 h fasting before tracer injection	Promotes myocardial utilization of fatty acids instead of glucose
**Physical activity** [95]	Avoid strenuous exercise before the examination	Prevents non-specific muscular FDG uptake
**Blood glucose control** [95]	PET/CT should be performed when blood glucose < 180 mg/dL	Reduces competitive inhibition of FDG uptake and improves image quality
**Insulin management** [95]	FDG injection should not occur within 6 h of short-acting insulin; long-acting insulin should be avoided on the day of the scan	Prevents increased myocardial glucose utilization and false-negative results
**Heparin administration** [95]	Optional IV unfractionated heparin (50 IU/kg) about 15 min before tracer injection	Further suppresses physiological myocardial uptake
**Image acquisition timing** [95]	PET/CT acquisition typically performed 60 min after FDG injection	Allows adequate tracer distribution and optimal lesion-to-background contrast
**Visual assessment of myocardial suppression** [95]	Myocardial FDG uptake compared with reference structures (descending aorta blood pool or liver)	Enables semiquantitative grading of myocardial suppression
**Semiquantitative grading (Swart scale)** [88].	Five-grade scale: Grade I (uptake lower than blood pool) to Grade V (uptake comparable to brain activity)	Standardized assessment of myocardial FDG suppression

Notes: PET/CT = positron emission tomography combined with computed tomography; FDG: ^18^F-fluorodeoxyglucose.

**Table 9:** Semiquantitative SUV thresholds obtained in FDG-PET/CT research studies of infective endocarditis.

Study	Clinical Setting	Parameter Evaluated	Proposed Threshold	Diagnostic Performance
**Swart et al.** [88]	Prosthetic valve endocarditis	SUV max	4.2	Sensitivity 60%, Specificity 91%
		SUV ratio	2.1	Sensitivity 75%, Specificity 86%
**Bensimhon et al.** [98]	CDRIE	SUV max	>2.2	Accurate discrimination of device pocket infection vs. controls
**Memmott et al.** [97]	CDRIE (dual-time-point imaging)	SUV max	2.90	Sensitivity 88%, Specificity 100%
		SUV ratio	1.63	Sensitivity 84%, Specificity 100%

Notes: CDRIE = cardiac device-related infective endocarditis.

**Table 10:** POET criteria for including EI patients.

Causative pathogen	Left-sided infective endocarditis with blood cultures positive for SA, CoNS, *Streptococcus* species, or Enterococcus faecalis.
Adequate hospital treatment	IV relevant antibiotics administered for ≥10 days and ≥7 days after heart surgery
Efficient response to IV antibiotic treatment	(-) No fever (T < 38 °C) > 2 days (-) CRP < 25% of the maximal measured value or absolute value < 20 mg/L (-) Leucocytes < 15 × 10^9^ per L during IV antibiotic treatment
Other indication for continued intravenous antibiotic administration	BMI > 40 kg/m^2^ or reduced gastrointestinal uptake
Oral antibiotic testing	bacterial susceptible examinations identify at least two different classes of orally administered antibiotics
Transesophageal echocardiogram	TEE was required to be performed at least twice: once before transition to oral therapy and once at the completion of antibiotic treatment

Notes. SA = *Staphylococcus aureus*; CoNS = coagulase-negative staphylococci; IV = intravenous; T = temperature; CRP = C-reactive protein; BMI = body mass index; L = liter; TEE = Transesophageal echocardiogram.

**Table 11:** Key unmet clinical needs and proposed multidisciplinary team approach.

Unmet Clinical Need	Clinical Challenge	Proposed Solution/Role of Interdisciplinary Approach
Uncertain diagnosis	Non-specific clinical presentation; limited sensitivity of single imaging modalities	Integration of multimodality imaging (TEE, CT, FDG-PET/CT) and microbiological techniques (PCR, serology); coordinated evaluation within an MDT; consideration of repeat imaging, particularly TTE
Identification of causative pathogen	Blood culture-negative IE; fungal or intracellular organisms	Appropriate blood culture timing and technique; use of molecular diagnostics (e.g., 16S rRNA PCR) and serology; collaboration between microbiologists and clinicians; awareness of local epidemiology
Risk stratification and timing of surgery	Difficulty in identifying patients at high risk of embolism or hemodynamic deterioration or long term prognosis	Application of validated risk scores; integration of clinical and imaging findings; shared decision-making within an MDT
Management of prosthetic valve and device-related IE	Diagnostic complexity due to artifacts; higher complication rates	Multimodality imaging (TEE, CT, FDG-PET/CT); collaboration between cardiologists, cardiac surgeons, and infectious disease specialists; appropriate patient preparation for imaging (e.g., FDG-PET/CT); systematic application of preventive strategies
Antimicrobial treatment optimization	Antimicrobial resistance; drug toxicity; prolonged therapy	Individualized antimicrobial strategies guided by infectious disease specialists and microbiology data, taking into account local epidemiology
Selection of candidates for OPAT or oral therapy	Risk of complications after early discharge; need for careful patient selection	Use of structured criteria (e.g., OPAT-GAMES, POET); multidisciplinary evaluation; consideration of comorbidities and availability of adequate social support
Variability in access to specialized care	Limited availability of MDTs and advanced imaging in some centers	Development of referral networks and tiered systems (primary, secondary, tertiary centers)
Patient adherence and follow-up	Long treatment duration; risk of relapse; poor adherence	Patient education; structured follow-up within an MDT; involvement of nurse specialists; tailored management in high-risk patients not undergoing surgery

Notes: TEE = transesophageal echocardiography; CT = computed tomography; FDG-PET/CT = ^18^F-fluorodeoxyglucose positron emission tomography combined with computed tomography; MDT = multidisciplinary team.

## Data Availability

Not applicable.

## Abbreviations

The following abbreviations are used in this manuscript:

2D	two-dimensional
3D	three-dimensional
CDRIE	cardiac device-related infective endocarditis
CIED	cardiac implantable electronic device
CRP	C-reactive protein
CoNS	coagulase-negative staphylococci
CT	computed tomography
DOAC	direct oral anticoagulant
ESC	European Society of Cardiology
FDG-PET/CT	^18^F-fluorodeoxyglucose positron emission tomography combined with computed tomography
HBAT	hospital-based antibiotic therapy
IDSA	Infectious Diseases Society of America
IE	infective endocarditis
ISCVID	International Society for Cardiovascular Infectious Diseases
IV	intravenous
MALDI-TOF MS	matrix-assisted laser desorption/ionization time-of-flight mass spectrometry
MDT	multidisciplinary team
MRSA	methicillin-resistant *Staphylococcus aureus*
MSSA	methicillin-sensitive *Staphylococcus aureus*
NVE	native valve infective endocarditis
OPAT	outpatient parenteral antibiotic therapy
PCR	polymerase chain reaction
PDTA	diagnostic and therapeutic care pathways
PVE	prosthetic valve endocarditis
ROC	receiver operating characteristic
SA	*Staphylococcus aureus*
SPECT	single photon emission computed tomography
spp	species
SUV	standardized uptake value
SUVmax	maximal standardized uptake value
TAVI	transcatheter aortic valve implantation
TEE	transesophageal echocardiography
TTE	transthoracic echocardiography
VWF	von Willebrand factor
